# Ruptured pancreaticoduodenal pseudoaneurysm causing arterioportal fistula: combined transarterial and transportal embolization

**DOI:** 10.1186/s42155-020-00165-8

**Published:** 2020-09-29

**Authors:** Nobuo Waguri, Rie Azumi, Akihiko Osaki, Munehiro Sato, Tsuneo Aiba, Koichi Furukawa

**Affiliations:** Department of Gastroenterology and Hepatology, Niigata City General Hospital, 467-3 Shumoku, Chuo-ku, Niigata, 950-1197 Japan

**Keywords:** Pseudoaneurysm, Arterioportal fistula, Pancreaticoduodenal artery, Transcatheter arterial embolization, Transportal embolization

## Abstract

**Background:**

Arterioportal fistulas are rare vascular disorders of the abdominal viscera. They are arteriovenous communications between the splanchnic arteries and the portal vein or its tributaries. We herein report a case of an extrahepatic arterioportal fistula that was caused by rupture of a pseudoaneurysm of the pancreaticoduodenal artery and successfully treated with embolization using a combination of the arterial and percutaneous transhepatic portal venous approaches.

**Case presentation:**

A 79-year-old man was transferred to our hospital because of the sudden appearance of a hematoma containing a large pseudoaneurysm in the mesentery of the duodenum. Emergency abdominal angiography revealed that a pseudoaneurysm of the anterior inferior pancreaticoduodenal arterial branch had perforated into the portal system (arterioportal fistula). We performed coil embolization via the inflow artery and portal vein using a percutaneous transhepatic approach. The patient recovered without complications and was discharged.

**Conclusion:**

This rare vascular disorder was successfully treated with an unplanned combination therapy. We believe that flexible strategy changes led to the successful treatment in this case.

## Background

Arterioportal fistulas (APFs) are rare vascular disorders of the abdominal viscera. They are arteriovenous communications between the splanchnic arteries and the portal vein or its tributaries. APFs are more commonly intrahepatic than extrahepatic (Vauthey et al. 1997). We herein report a case of an extrahepatic APF that was caused by rupture of a pseudoaneurysm of the pancreaticoduodenal artery and successfully treated with embolization using a combination of the arterial and percutaneous transhepatic portal venous approaches.

## Case presentation

**Fig. 1:**
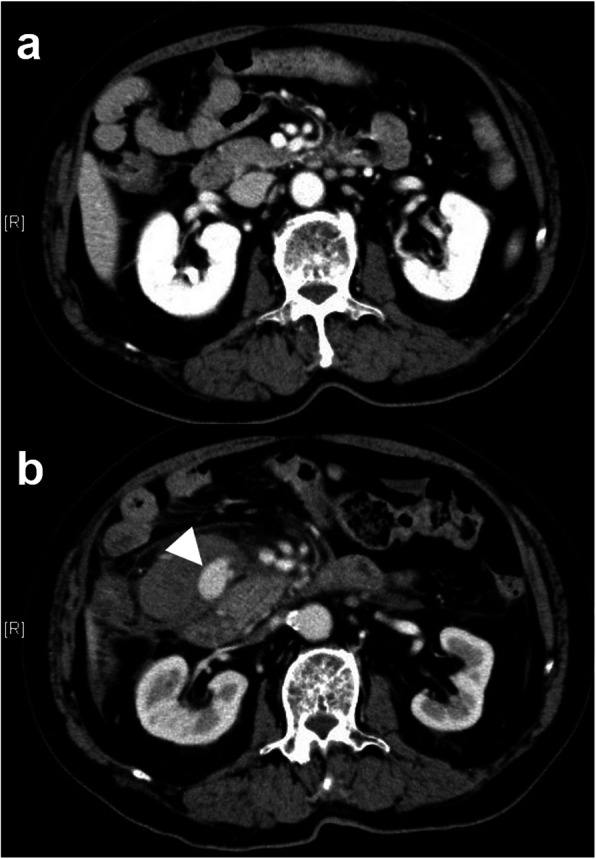
Contrast-enhanced computed tomography (CT) findings at the previous hospital. **a** CT examination at admission to the previous hospital showed no abnormal findings around the superior mesenteric artery and vein or the duodenum. **b** CT images obtained on the fourth day after symptom onset demonstrated a newly developed hematoma with a pseudoaneurysm (white arrowhead) in the mesentery of the duodenum

A 79-year-old male tourist was admitted to a previous hospital with a fever of 38.4 °C and severe nausea. Because of elevated hepatobiliary enzymes (aspartate aminotransferase, 290 U/L; alanine aminotransferase, 349 U/L; and gamma-glutamyl transpeptidase, 511 U/L), mild jaundice (total bilirubin, 4.8 mg/dL), and gallbladder stones on ultrasonography, he was diagnosed with cholangitis and treated with antibiotics. Computed tomography (CT) on the day of admission to the previous hospital (day 1) revealed no ileus, bile duct obstruction, or dilation, and the cause of the symptoms could not be identified. However, the nausea persisted, and abdominal fullness suddenly appeared on day 4. CT on day 4 demonstrated a newly developed hematoma with a diameter of 72 mm and a pseudoaneurysm with a diameter of 24 mm that had not been seen on day 1 in the mesentery of the duodenum (Fig. 1a, b). Therefore, the patient was transferred to our hospital for treatment. Upon admission to our hospital, laboratory tests including measurement of routine hematology and blood chemistry parameters showed slight anemia and elevated hepatobiliary enzymes (Table 1). After we had discussed the patient’s case with the surgeons, we decided to prioritize interventional radiology (IR) treatment because the pseudoaneurysm could be more accurately approached by angiography than laparotomy. The patient provided written consent for angiography and IR.

**Table 1:** Laboratory findings on admission

White blood cell count (/μL)	7300
Red blood cell count (10^6^/μL)	3.37
Hemoglobin (g/dL)	10.3
Hematocrit (%)	31.8
Platelet count (10^3^/μL)	131
Prothrombin time (international normalized ratio)	0.89
Fibrinogen (mg/dL)	367
Fibrin degradation products (μg/mL)	6.0
Total protein (g/dL)	6.7
Albumin (g/dL)	3.8
Aspartate aminotransferase (U/L)	62
Alanine aminotransferase (U/L)	129
Lactate dehydrogenase (U/L)	207
Alkaline phosphatase (U/L)	201
Gamma-glutamyl transpeptidase (U/L)	278
Total bilirubin (mg/dL)	1.4
Direct bilirubin (mg/dL)	0.3
Creatinine kinase (U/L)	338
Amylase (U/L)	61
Lipase (U/L)	68
Blood urea nitrogen (mg/dL)	14.0
Creatinine (mg/dL)	0.60
C-reactive protein (mg/dL)	1.74

**Fig. 2:**
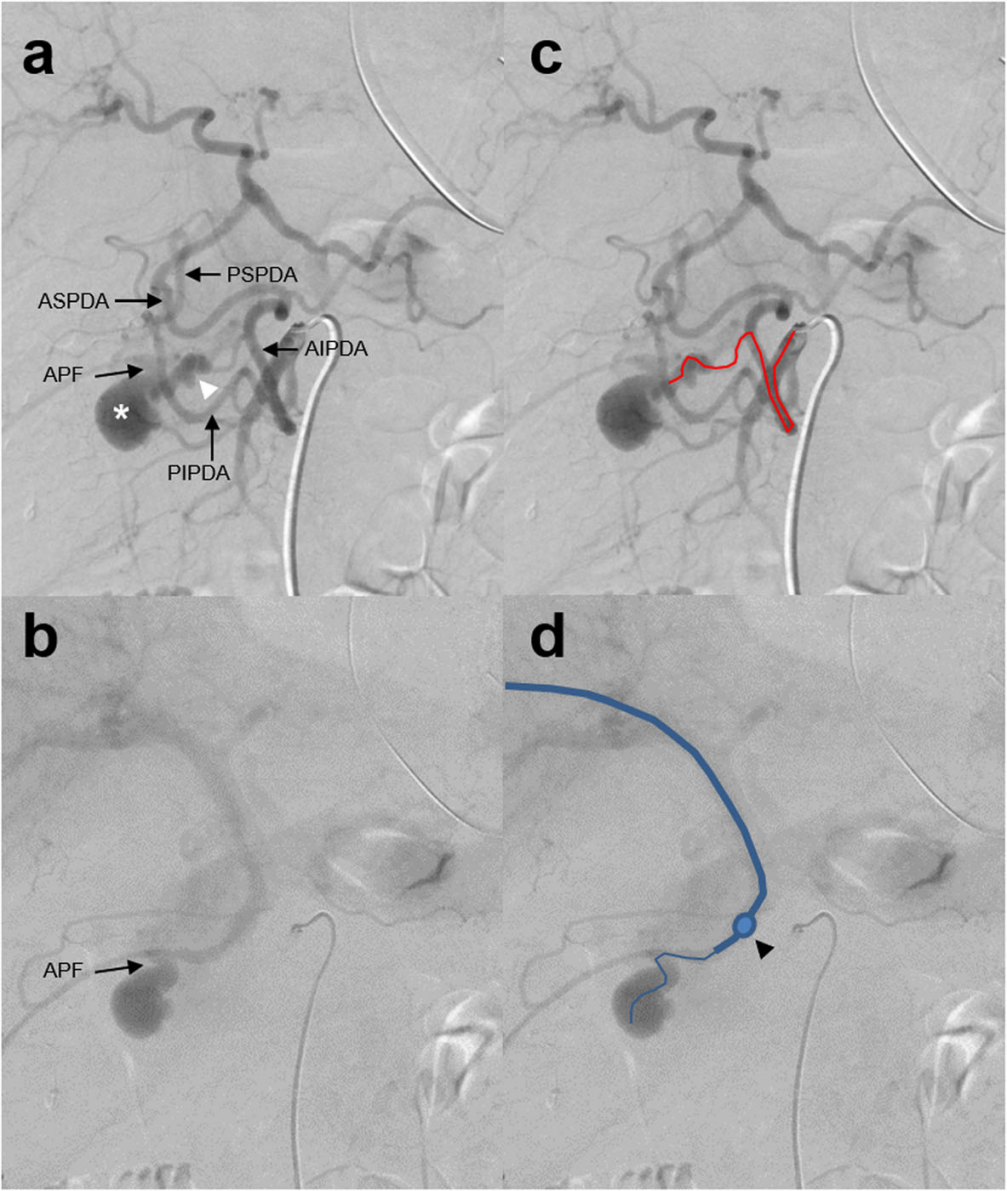
Emergency angiography findings: left-anterior oblique 20-degree images of superior mesenteric arteriography. **a** The hepatic and splenic arteries were viewed via the pancreaticoduodenal arcade, and the routes from the anterior and posterior inferior pancreaticoduodenal arteries (AIPDA and PIPDA) to the anterior and posterior superior pancreaticoduodenal arteries (ASPDA and PSPDA) were observed. No extravasation was present. A pseudoaneurysm (white asterisk) was visualized via the branch of the AIPDA (white arrowhead). The arcade from the PIPDA to the PSPDA was not involved in the pseudoaneurysm. **b** The portal vein was subsequently visualized from the pseudoaneurysm through the arterioportal fistula (APF). **c** Same image as a). The trace of the microcatheter advanced via the AIPDA is shown by the red line. **d** Same image as b). A balloon catheter was advanced to the hepatic side of the APF using a percutaneous transhepatic portal approach, and the microcatheter was advanced through the balloon catheter to the pseudoaneurysm (blue line). The position of the balloon is indicated by a black arrowhead

**Fig. 3:**
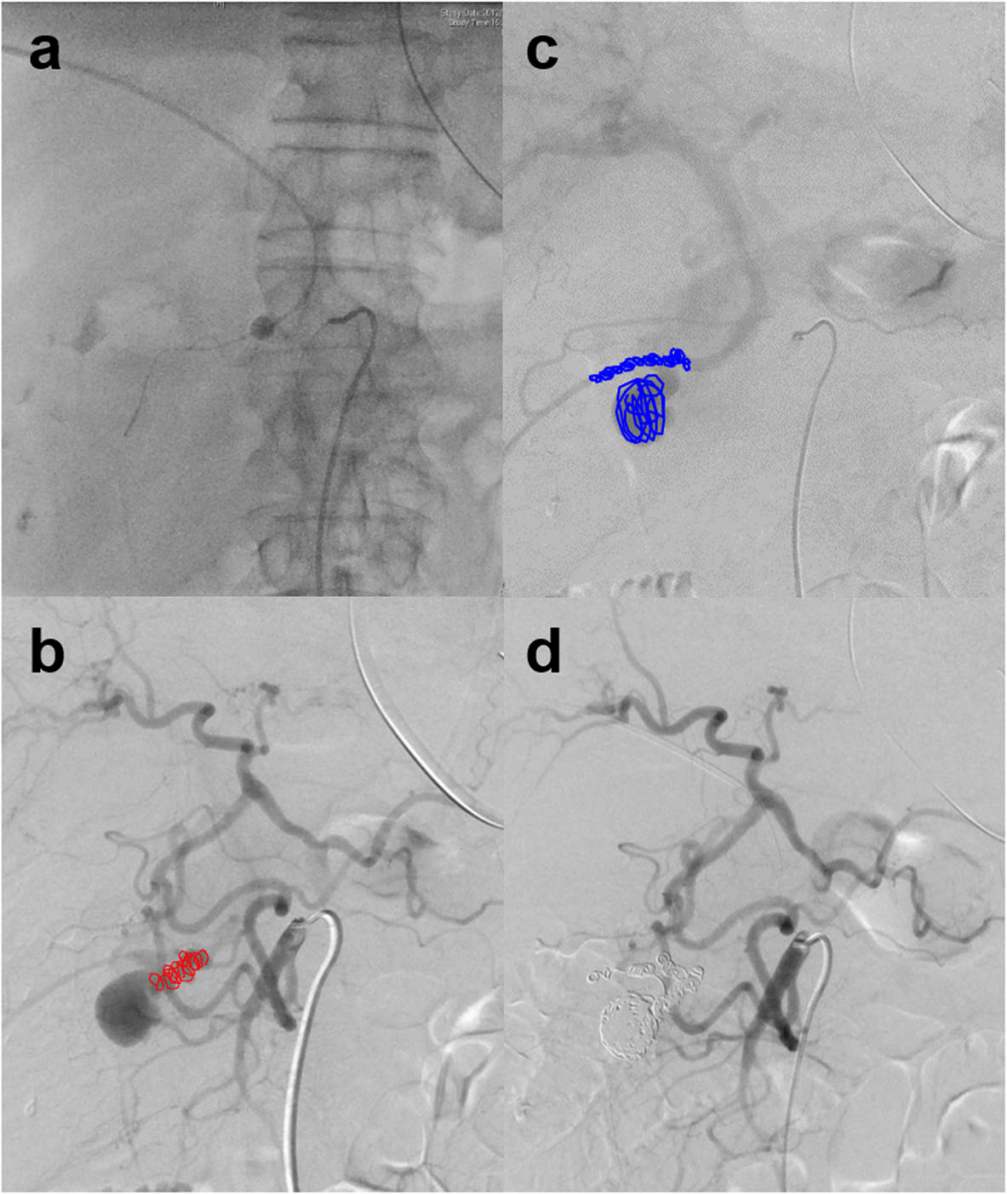
Emergency angiography and interventional radiology. **a** The catheters were advanced via the artery and the portal vein. **b** Left-anterior oblique 20-degree image of the arterial phase of superior mesenteric arteriography. The embolic microcoils placed from the arterial catheter to the inflow branch of the pseudoaneurysm are shown in red. **c** An image of the portal phase following Fig. 3b. The embolic microcoils placed in the pseudoaneurysm and outflow vessel from the transportal catheter are shown in blue. **d** Superior mesenteric arteriography demonstrating the disappearance of blood flow to the pseudoaneurysm after coil embolization using a combination of the arterial and percutaneous transhepatic portal venous approaches

Emergency angiography was performed to treat the pseudoaneurysm that had suddenly appeared and ruptured. Superior mesenteric arteriography revealed the hepatic and splenic arteries through the pancreaticoduodenal arcade. No extravasation was observed, but a pseudoaneurysm of the anterior inferior pancreaticoduodenal arterial branch and early visualization of the portal vein was found (Fig. 2a, b). We diagnosed an APF caused by rupture of a pseudoaneurysm. We could not advance a 1.98-French microcatheter (Parkway Soft; Asahi Intecc, Seto, Japan) to the APF or even to the pseudoaneurysm because the artery was narrow and meandering (Fig. 2c). Thus, transcatheter arterial embolization (TAE) using fibered embolization microcoils (Tornado Embolization Coil; Cook Medical, Bloomington, IN, USA) could only be performed upstream of the pseudoaneurysm. Transportal embolization with the percutaneous transhepatic approach was subsequently added. The right anterior superior subsegmental branch of the intrahepatic portal vein (P8) was punctured under ultrasonographic guidance, and a 5-French angio-sheath was inserted into the portal trunk. A 5.2-French catheter with an occlusive balloon (9-mm diameter, Selecon MP Catheter; Terumo Clinical Supply, Gifu, Japan) was placed at the outflow tract of the APF. A 1.98-French microcatheter (Parkway Soft; Asahi Intecc) was then introduced into the pseudoaneurysm through the APF in a retrograde fashion (Figs. 2d, 3a). We performed coil placement inside the pseudoaneurysm as a foothold and embolization of the outflow portal branch using microcoils (IDC-18 and Interlock Fibered IDC; Boston Scientific, Marlborough, MA, USA and Tornado embolization coil; Cook Medical) under the flow control by balloon occlusion (Fig. 3b). From the arterial side, the inflow vessel of the pseudoaneurysm was coil-embolized by the usual TAE technique (Fig. 3c). As a result, embolization of the inflow and outflow tracts of the pseudoaneurysm was achieved (Fig. 3d). The portal vein catheter was removed while filling the puncture route of the liver parenchyma with gelatin sponge foam. No coil migration or elevation of hepatobiliary enzymes occurred postoperatively. The patient recovered without complications and was discharged to his place of residence 20 days after IR.

## Discussion

The most common cause of APFs is trauma including blunt trauma, stab wounds, and gunshot wounds, followed by iatrogenic causes such as surgical and nonsurgical interventions, congenital abnormalities, tumors, and aneurysms (Vauthey et al. 1997). Our patient was diagnosed with an acquired extrahepatic APF caused by rupture of an anterior inferior pancreaticoduodenal arterial pseudoaneurysm. The pseudoaneurysm developed suddenly and ruptured within 4 days. The cause of pseudoaneurysm formation was presumed to be cholangitis, which had triggered the admission. The tentative diagnosis of cholangitis was based on the presence of gallstones, elevation of hepatobiliary enzymes, and a mild inflammatory response. However, CT showed neither bile duct dilatation nor edematous thickening of the bile duct wall. Thus, whether the cholangitis caused enough inflammation to form the pseudoaneurysm in this case remains questionable. The rupture of the pseudoaneurysm resulted in a hematoma in the mesentery and perforation of the portal tributary. We have herein reported our experience from onset to treatment of this rare condition.

Patients with APFs may be asymptomatic or symptomatic with complications of portal hypertension (ascites, diarrhea, and gastrointestinal bleeding), congestive heart failure, and intestinal ischemia due to the blood steal phenomenon (Vauthey et al. 1997; Al-Khayat et al. 2008). This case of an acquired large extrahepatic APF corresponds to type 2 in the classification of APFs established by Guzman et al. (2006). Type 2 APFs have large shunt flow and are therefore likely to cause symptoms of portal hypertension. The symptoms associated with APFs depend on the location and size of the fistula (i.e., shunt flow). However, these symptoms do not develop immediately after APFs formation. In this case, the rupture of the pseudoaneurysm was urgently treated, so no symptoms of portal hypertension after APF formation were seen.

The main treatment for APFs was surgery (Vauthey et al. 1997); in recent years, however, IR has become mainstream. This technique allows treatment while confirming the fistula site by arteriography. Simple TAE (Siablis et al. 2006; Marrone et al. 2006; Yamazaki et al. 2017) is indicated at sites where collateral blood flow to the basin is maintained and organ ischemia cannot occur. Because covered stent treatment can block the fistula while preserving the main arterial blood flow, it is adopted in relatively large arterial regions such as the common hepatic artery, gastroduodenal artery (Krishan et al. 2010), splenic artery, and superior mesenteric artery trunk (Narayanan et al. 2008; Yeo et al. 2008). If a pseudoaneurysm or fistula exists in the main arcade of the pancreaticoduodenal artery, stent treatment may be considered. In this case, however, stent placement was not appropriate because both a pseudoaneurysm and APF were present in a branch that diverged from the mainstream of the arcade, and even a microcatheter could not reach this site. Some reports have described successful treatment of an APF using transportal embolization after TAE was unsuccessful or inadequate (Denys et al. 1998; Cekirge et al. 1998; Wada et al. 2016). In this case, we embolized the inflow of the pseudoaneurysm with TAE and the outflow from the APF with transportal embolization. The outflow was blocked with a balloon catheter to prevent the microcoils from flowing out to the portal vein. Because one report described a case in which the coil for TAE flowed to the portal vein through the APF (Weinstein et al. 2009), it seems important to back up the portal vein side with a balloon. A pseudoaneurysm should not be densely packed because it does not have a wall structure and is easily ruptured by coil filling. In this case, the microcatheter via the portal vein could be advanced beyond the APF to the pseudoaneurysm; thus, coil embolization was started from within the pseudoaneurysm as a foothold to avoid dense packing. We added embolization to the outflow vessels of the portal side. As a result, the blood flow of the pseudoaneurysm was completely controlled.

In an emergency, necessary and sufficient treatment can save lives even if the treatment is slightly overzealous. Even in cases of difficult treatment, an accurate understanding of the present situation and a flexible response will lead to success.

## Conclusions

In summary, this case shows that rupture of a pseudoaneurysm can cause an APF and that embolization from both the artery and portal vein is effective. No previous reports have described embolization from both the artery and portal vein for treatment of an APF caused by a ruptured pseudoaneurysm of the pancreaticoduodenal artery. We believe that such flexible strategy changes led to the successful treatment in this case.

## Data Availability

The datasets used and/or analyzed during the current study are available from the corresponding author on reasonable request.

## Abbreviations

APF: Arterioportal fistula
CT: Computed tomography
IR: Interventional radiology
TAE: Transcatheter arterial embolization
